# Cardiac and Autonomic Dysfunctions Assessed Through Recurrence Quantitative Analysis of Electrocardiogram Signals and an Application to the 6-Hydroxydopamine Parkinson’s Disease Animal Model

**DOI:** 10.3389/fphys.2021.725218

**Published:** 2021-11-24

**Authors:** Lucas Shinoda, Laís Damasceno, Leandro Freitas, Ruy Campos, Sergio Cravo, Carla A. Scorza, Fúlvio A. Scorza, Jean Faber

**Affiliations:** ^1^Neuroscience Division, Department of Neurology and Neurosurgery, Escola Paulista de Medicina, Federal University of São Paulo, São Paulo, Brazil; ^2^Cardiovascular Division, Department of Physiology, Escola Paulista de Medicina, Federal University of São Paulo, São Paulo, Brazil; ^3^Nucleus of Neuroengineering and Computation, Institute of Science and Technology, Federal University of São Paulo, São Paulo, Brazil

**Keywords:** recurrence quantitative analysis, Poincaré map, Parkinson’s disease, computational ECG model, 6-OHDA animal model, HRV (heart rate variability) and ECG-complexes, cardiac and autonomic dysfunctions

## Abstract

A classic method to evaluate autonomic dysfunction is through the evaluation of heart rate variability (HRV). HRV provides a series of coefficients, such as Standard Deviation of n-n intervals (SDNN) and Root Mean Square of Successive Differences (RMSSD), which have well-established physiological associations. However, using only electrocardiogram (ECG) signals, it is difficult to identify proper autonomic activity, and the standard techniques are not sensitive and robust enough to distinguish pure autonomic modulation in heart dynamics from cardiac dysfunctions. In this proof-of-concept study we propose the use of Poincaré mapping and Recurrence Quantification Analysis (RQA) to identify and characterize stochasticity and chaoticity dynamics in ECG recordings. By applying these non-linear techniques in the ECG signals recorded from a set of Parkinson’s disease (PD) animal model 6-hydroxydopamine (6-OHDA), we showed that they present less variability in long time epochs and more stochasticity in short-time epochs, in their autonomic dynamics, when compared with those of the sham group. These results suggest that PD animal models present more “rigid heart rate” associated with “trembling ECG” and bradycardia, which are direct expressions of Parkinsonian symptoms. We also compared the RQA factors calculated from the ECG of animal models using four computational ECG signals under different noise and autonomic modulatory conditions, emulating the main ECG features of atrial fibrillation and QT-long syndrome.

## Introduction

The autonomic nervous system (ANS) modulates cardiovascular function *via* two main pathways, the sympathetic (SNS) and parasympathetic (PNS) systems that play agonist-antagonist roles (Cannon, 1939). In general, SNS activation increases the heart rate and cardiac ventricle contractility, while the PNS mainly decreases heart rate, with faster local responses in the atrium transmitted *via* the vagus nerve (Hopkins and Armour, 1984; Shivkumar et al., 2016). Since autonomic balance modulates the heart rate (Sztajzel, 2004), the simplest way to analyze ANS activity is to measure heart rate variability (HRV) using ECG recordings. Traditional techniques quantify autonomic modulations searching for frequency characteristics, namely, low frequency (LF), high frequency (HF), and their ratio LF/HF ranges or temporal features, standard deviation of NN intervals (SDNN), and root mean square of successive R-R interval differences (RMSSD), on the ECG-*tachogram* along the time series constructed (most commonly) from the R-R peak time distances (RR: interbeat intervals between all successive heartbeats; NN: interbeat intervals from which artifacts have been removed) (Malliani et al., 1994; Montano et al., 2009; Akselrod et al., 2014). However, these techniques are limited and require assumptions that are difficult to verify, especially for small ECG samples, which make these quantifications unreliable (Mansier et al., 1996). Some studies have used non-linear techniques such as Poincaré Map (PM) (or First Return map) and Recurrence Quantification Analysis (RQA) to find other ANS and ECG characteristics associated with heart and ANS disorders (Kamen and Tonkin, 1995; Tulppo et al., 1997). These methods have already been applied to ANS dysfunction related to seizures and sudden death, revealing their capacity to characterize biosignals in a clinical context (Zbilut and Webber, 1992; Marwan et al., 2002; Marwan, 2003; Billeci et al., 2018; Khazaei et al., 2018).

To assess the autonomic dysfunction associated with heart rate dynamics, we propose a proof-of-concept study where we constructed a set of four artificial ECG patterns modeling the main ECG features related to the two most common autonomic-cardiac dysfunctions, atrial fibrillation (AF) and long-QT syndrome (QT), and two control ECG signals, a complete periodic regular ECG (DET) activity without noise, and an ECG pattern with high Gaussian noise (GN). The AF and long-QT syndrome patterns were chosen mainly because they are very prevalent in Parkinson’s disease (PD) (Tysnes and Storstein, 2017).

Parkinson’s disease is a neurodegenerative disorder characterized by decreased levels of dopamine in the *striatum* and *substantia nigra* (Stephen et al., 1988; Poewe et al., 2017). Although it is mainly characterized by motor manifestations, non-motor conditions often precede motor symptoms (Braak et al., 2003; Schapira et al., 2017). Autonomic dysfunction (AD) is diagnosed in 80% of patients with PD, and can be aggravated due to a denervation of autonomic pathways, causing orthostatic hypotension and cardiac autonomic imbalance (Orimo et al., 1999; Smit et al., 1999; Goldstein et al., 2000; Goldstein, 2006; Evatt et al., 2009; Velseboer et al., 2011; Schapira et al., 2017). In this way, finding a robust and sensitive quantitative technique that can perform a better characterization of possible electrophysiological biomarkers from ECG signals may represent a paradigm shift in the diagnosis and progression monitoring of this disease (van Dijk et al., 1993; Cersosimo and Benarroch, 2013).

Through the Poincaré map and RQA factors relative to the four artificial ECG patterns, we were able to characterize and identify the main non-linear ECG and HRV features associated with the AR and QT disorders (Rodrigues et al., 2019). We then applied the Poincaré map and RQA techniques on ECG recordings from a small set of animal models of Parkinson’s disease, using the unilateral 6-hydroxydopamine (6-OHDA) model that, with lesions of the nigrostriatal pathway, produce similar motor impairments to those seen in PD and a sham group (Ungerstedt, 1968). By projecting their non-linear factors on the artificial ECG factors, we were able to compare them with the same non-linear features assessed in the ECG recordings of the PD animal models. This comparison produces a systematic protocol for better physiological interpretation and validation of these techniques considering the PD autonomic-heart dysfunction scenario.

## Materials and Methods

All experiments were approved by the Animal Care and Use Committee of the Federal University of São Paulo (protocol: CAAE 6463110417), and the analysis applied to biological signals was approved by the Ethics and Research Committee of the Federal University of São Paulo, under the protocol number CAAE 7299310719.

To study the possible effects associated with dysautonomia, we considered three different approaches: (i) PRQRST complex analysis focusing on the waveform characteristics, such as their amplitude variations; (ii) HRV analysis by evaluating the tachogram characteristics through their Poincaré Maps; and (iii) ECG signal analysis by evaluating their non-linear dynamics through RQA.

To perform the RQA analysis, we built four simple distinct artificial ECG signals (aECG), each with a predominant feature: (1) deterministic (DET), (2) atrial fibrillation (AF), (3) long QT syndrome (LQT), and (4) Gaussian noise (GN). We then calculated the low-frequency and high-frequency ratio (LF/HF) modulation from the R-R tachogram patterns of each aECG. Finally, we added white noise with different intensities to the R-R tachograms to simulate different degrees of dysautonomia most prevalent in PD.

Furthermore, we consider the application of these techniques to a set of ECG signals recorded from Wistar rats using the unilateral 6-hydroxidopamine (6-OHDA) model to mimic PD based on the Ungerstedt protocol (Ungerstedt, 1968). Two independent groups of rats were studied: the experimental group (6-OHDA, *n* = 3) and the control group (Sham, *n* = 3), which underwent the same surgical procedure as the 6-OHDA set, free of any drugs. We performed an exhaustive search in both sets of ECG signals looking for epochs where the standard ECG and HRV metrics could not distinguish 6-OHDA features from Sham features, but RQA could. Finally, the ECG and HRV features described by the four aECG patterns, using different quantification techniques, were compared with ECG signals recorded from 6-OHDA and from sham animal models to identify similarities between 6-OHDA models with AF, LQT, DET, and GN, and establish a better signal interpretation.

### Artificial Electrocardiogram Model

To correlate the information from RQA factors with PD conditions, we implement four distinct computational models of ECG (aECG), mimicking four special heart conditions that are most prevalent in PD. These artificial ECG signals were based on the McSharry model (Mcsharry et al., 2003), which in turn is based on the tachogram power spectrum and calculated from the R-R peak intervals of the aECG signals. The tachograms were built using a heart rate of 350 bpm with a standard deviation of 50 bpm to represent the standard dynamics of rat heartbeats.

By varying the low and high frequency ratio (LF/HF), the tachograms reflected the temporal distribution of the aECG complexes over time. In this way, by applying the inverse Fourier transform to the LF/HF, the power spectrum of the tachogram is extracted, and the (artificial) ECG signal is reconstructed by sequentially introducing a standard PQRST complex model. The standard PQRST complexes were adapted to fit the physiological patterns of a rat animal model (increasing HR and removing Q waves), since the standard PQRST complex was designed for human heart ECG (Mcsharry et al., 2003). Gaussian noise was added considering 1% of the amplitude of the aECG signals to describe more realistic patterns.

The aECG patterns were constructed to represent the following four conditions: (1) a regular, deterministic ECG signal (DET) without extra noise or any other effect to be used as a control; (2) atrial fibrillation ECG signal (AF), built by removing the P waves and replacing them with white noise along the PQ interval; (3) long QT syndrome (LQT), built by stretching the QT interval in time and decreasing its amplitude; and (4) a noisy ECG signal (NSE), created by introducing a high-level white noise pattern, with 100% amplitude on the DET aECG pattern. The aECGs (1) and (4) were used as control signals while the aECGs (2) and (3) were used to represent the heart dysfunction models described in the literature (Oka et al., 1997; Deguchi et al., 2002; Çanga et al., 2018). Finally, for each aECG model, four noise degrees were added to their corresponding tachograms, 0, 33, 66, and 99% of maximum amplitude, and four different modulation levels of the LF/HF ratio, given by 0, 0.5, 1, 1.5, to represent different autonomic balance effects on heart dynamics. Using the McSharry model (Mcsharry et al., 2003) we adopted:

(1) DET signal was the default model: angles *θ*_i_ (degree) [P: −70, Q: −15, R: 0, S: 15, T: 100]; *a*_i_ [P: 1.2, Q: −5, R: 30, S: −7.5, T: 0.75]; *b*_*i*_ [P: 0.25, Q: 0.1, R: 0.1, S:0.1, T: 0.4];
(2) LQT signal (long QT waves—it did not emulate arrythmia): *θ*_i_ [P: −70, Q: −15, R: 0, S: 15, T: 100]; *a*_i_ [P: 1.2, Q: −5, R: 30, S: −7.5, T: 0.75]; *b*_i_ [P: 0.25, Q: 0.1, R: 0.1, S: 0.1, T: 0.6];
(3) AF signal (low P-wave peak): *θ*_i_ [P: −70, Q: −15, R: 0, S: 15, T: 100]; *a*_i_ [P: 0.2, Q: −5, R: 30, S: −7.5, T: 0.75]; *b*_i_ [P: 0.125, Q: 0.1, R: 0.1, S:0.1, T: 0.4];
(4) NSE signal = (100% of gaussian noise) × DET;

### Rat Electrocardiogram

Rat ECG signals were registered from a 6-hydroxydopamine (6-OHDA) animal model based on the procedure described by Ungerstedt (1968). This model is based on the degradation of dopamine neurons in the substantia nigra, mimicking the PD condition. The sham control group received the same surgical procedure, but without the addition of 6-OHDA in the brain.

We used a total of six Wistar rats, *n* = 3 6-OHDA and *n* = 3 sham, weighing 230–300 g from the experimental animal center of the Federal University of São Paulo—UNIFESP, maintained at a temperature of 21° ± 2°C, and light and dark cycle of 12 h with free access to food and water (for more details see Rodrigues et al., 2019).

All ECG data were recorded using PowerLab 8/35 (Adinstruments, Australia) and recorded at a sampling rate of 1,000 Hz for 60 min on day 14. The process and visualization methods were performed using MATLAB^TM^ software and a computer with 8 GB RAM, Intel^®^ Core^TM^ i7-6700 processor, 3.4 GHz.

To perform the analysis, all ECG signals were recorded for approximately 2 h. Due to animal movements and environmental interference, we opted for a conservative selection epoch, considering 15 window samples of 20 s sparsely (Acharya et al., 2006).

### Electrocardiogram Waveform Analysis

The waveform analysis applied to the PQRST complexes was based on Quiroga Spike Sorting (Quiroga, 2012). In this technique, the main signal changes for a specific event are searched via principal component analysis (PCA) decomposition. In this approach, the PCA features correspond to the associated time window of each PQRST complex, aligned through the R-peaks as a reference, with an interval of 18 ms for both sides. If their waveforms covariate along the ECG complexes, clusters will appear, emphasizing differences and similarities.

### Standard Heart Rate Variability Features

**FIGURE 1:**
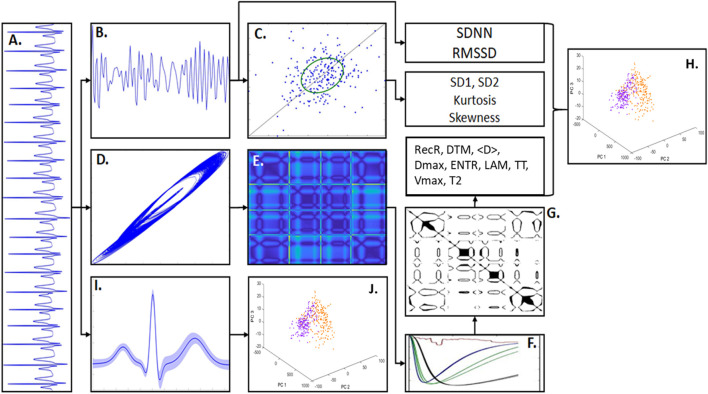
Methodology scheme. **(A)** An example of artificial ECG pattern. By quantifying the R-R intervals from the ECG signals it was built the correspondent tachogram time-series consisted of R-R sequences. **(B)** From the ECG tachogram it was quantified two temporal features, standard deviation of n-n intervals (SDNN) and root mean square of successive differences (RMSSD) and built the Poincaré Map (PM). **(C)** The PM represents the scatter of points from the tachogram-space with the axis (RR_n,_ RR_*n*+τ_). The phase spaces of the ECG signals were also constructed, given by the coordinates (X_*t*_, X_t+τ_, X_t+2τ_), which shows the signal trajectory seen in **(D)**. In **(E)** is shown the distance matrix, representing the Euclidian distance of each sample-point in relation to all other points in the phase-space. **(F)** Shows the recurrence quantification analysis (RQA) characteristics that allowed to select an epsilon threshold. **(G)** Shows an example of Recurrence Plot from the distance matrix given an epsilon. The RQA factors calculated in this work were: recurrence rate (RecR), determinism (DTM), mean diagonal length (<D>), maximum diagonal length (Dmax), Shannon entropy (ENTR), Trapping Time (TT), laminarity (LAM), maximum vertical length (Vmax), and time type 2 (T2). **(H)** Principal component analysis (PCA) calculated from the RQA factors highlights the differences between PD model and sham groups. **(I)** ECG waveform samples (PQRST complexes) extracted from the ECG patterns (considering its mean and SD). **(J)** PCA calculated from the ECG waveforms highlights the main differences in the signal morphology.

To evaluate differences in the autonomic balance activity in each ECG group, two main features of the heart rate variability (HRV) were calculated from their tachograms, namely, standard deviation of NN intervals (which are the standard deviation of normal intervals of RR, disregarding ectopic beat, known as SDNN) and root mean square of successive differences (RMSSD). The SDNN describes the autonomic balance for long periods of time, whereas RMSSD provides autonomic balance data for short periods of time (Shaffer and Ginsberg, 2017) (see Figures 1A,B).

### Poincaré Map (First Return Map)

We also evaluated the autonomic balance using Poincaré Maps, which consists of scatter plots given by past R-R intervals (RR*_*t*–1_*) against present R-R intervals (RR*_*t*_*) (Brennan et al., 2001). Through Poincaré maps, it is possible to characterize the tachograms according to their scatter patterns (Woo et al., 1992). This is done qualitatively by studying cluster shapes and quantitatively using the deviations SD2 and SD1, which represent the major axis and the minor axis of an ellipse, respectively (see Figure 1C). The standard deviation SD2 quantifies the point distribution across the line of identity (LOI), and the standard deviation SD1 quantifies the point distribution across the line perpendicular to the LOI. Both are directly associated with ECG beat-to-beat interval distribution (Brennan et al., 2001) and autonomic balance. SD1 is strongly correlated with LF, which represents parasympathetic modulation (Shaffer and Ginsberg, 2017).

### Recurrence Quantification Analysis

To evaluate the non-linear ECG features, we applied RQA. RQA is a technique applicable to any type of time series that allows access to time series characteristics that standard techniques cannot (Eckmann et al., 1995). Through RQA coefficients, it is possible to categorize a signal according to its level of stochasticity, chaoticity, and determinism, which can help to understand the type of physical coupling under its dynamics (Marwan et al., 2002). RQA is based on signal phase-space reconstruction, defined by


(1) $$\begin{array}{c} X:X(x_{0},x_{1},x_{2},\ldots ,x_{\tau_{m-1}};x_{1},x_{2},x_{3},\ldots ,x_{m};x_{0},x_{1},\\ x_{2},\ldots ,x_{m}) \end{array}$$

where τ is a parameter time delay, and the dimension *m* is a dimensionality parameter. Any time series can be described through its phase space in a two-dimensional matrix, representing the distances from every sample point. Additionally, a threshold ε must be defined, limiting which higher values are defined as recurrences, creating a recurrence plot (RP), defined by:


(2) $$RP\left(i,j\right)=H\left(\varepsilon -DM\left(i,j\right)\right),\left\{ \begin{array}{c} H>0\Rightarrow RP\left(i,j\right)=1\\ H<0\Rightarrow RP\left(i,j\right)=0 \end{array}\right.$$

where H(.) is the Heaviside step function and DM(.) is the distance matrix that contains all point-to-point Euclidian distances in the phase space (Marwan et al., 2007) (Figures 1D,E).

All parameters, τ and m and ε, were calculated individually for each signal, following the statistical characteristics of each one. After, using the Sturges Formula to optimize the histogram bins, the mutual information was determined (Sturges, 1926). Thus, for each signal, the chosen value of τ was the one that maximize the time-lag of 10 window samples of 20 s and that minimize the mutual information lower than 1/e (Kantz and Schreiber, 1997; Marwan and Webber, 2015). The dimension *m* was calculated using the False Nearest Neighbors (FNN) technique, considering the 10 window samples with the predefined tau. Following previous studies, the chosen value of m corresponded to the first dimension that present a FNN less than 0.1 (Fraser and Swinney, 1986; Zbilut and Webber, 1992; Marwan, 2003). Finally, the value of ε is based on the maximum phase-space diameter percentage, using the Euclidian distance, as described in Marwan et al. (2007). That is, we chose heuristically ε = 9%, from an interval of 1–15% of the maximum diameter associated with the phase space, given a value of τ and *m* that maximized the differences between the groups of interest. Additionally, the values in *DM*(*i*,*j*) were normalized by the maximum diameter of the phase space describing all data at the same scale (see Figures 1F,G).

To quantify the ECG signal recurrences, we chose nine RQA coefficients calculated on the dot structure patterns displayed by the recurrent plots (RPs) (Marwan et al., 2007). Only structures with more than three dots were considered as a proper recurrence, minimizing spurious effects. All the coefficients are based on the percentage of dots, horizontal dot-line structures, and diagonal dot-line structures on RPs, where each one provides information associated with time recurrences and trajectories according to the degree of stochasticity, chaoticity, or determinism (Zbilut and Webber, 1992; Gao, 1999; Marwan et al., 2002, 2007; Marwan, 2003). The nine coefficients used in this study are defined as follows:

*Recurrence rate (RecR)* quantifies the density of recurrences. This indicates the regularity of signal recurrences in the signal. Therefore, it is associated with the determinism.


(3) $$RecR\left(\varepsilon ,N\right)=\frac{1}{N^{2}-N}\sum_{i\neq j=1}^{N}RP_{i,j}^{m,\varepsilon }$$

where ε is the threshold, *N* is the total number of elements in RP, and $RP_{i,j}^{m,\varepsilon }$ are the RP *i,j*-elements, calculated using Equation (1), according to the threshold ε and embedded in dimension *m*.

*DTM* quantifies the density of recurrence time intervals. This indicates the regularity in the signal. It is defined by:


(4) $$DTM=\frac{\sum_{d=d_{min}}^{N}dP_{d}\left(d\right)}{\sum_{d=d_{min}}^{N}dRP_{i,j}}$$

where *P*_*d*_(.) is the probability of finding a diagonal with length *d*, calculated from a histogram, and *d*_*min*_ is the minimum acceptable distance value (defined as three dots).

*The average diagonal length (<D>)* quantifies the weighted average length of time recurrence. This indicates divergence of the space-phase trajectory; therefore, it is associated with stochasticity.


(5) $$<D>=\frac{\sum_{d=d_{min}}^{N}dP_{d}\left(d\right)}{\sum_{d=d_{min}}^{N}P_{d}\left(d\right)}$$

*The maximum diagonal length (Dmax)* quantifies the maximum length of a time recurrence. When inverted, it indicates the exponential divergence of the phase-space trajectory.


(6) $$D_{max}=\text{max}\left(d\right)$$

*The Shannon entropy (ENTR)* quantifies the complexity of interval recurrences. This indicates the stochasticity and chaoticity of the signal:


(7) $$ENTR=-\sum_{d=d_{min}}^{N}P_{d}\left(d\right)\ln P_{d}\left(d\right)$$

where *P*_*d*_(.) is the probability of obtaining the diagonal with length *d* in the diagonal length of the RP, and *ln(.)* is the natural logarithm of *P*_*d*_(*d*).

*Laminarity (LAM)* quantifies the percentage of fixed events in each time interval of recurrences. Since it evaluates the relative number of laminar events, it is associated with chaoticity:


(8) $$LAM=\frac{\sum_{v=v_{min}}^{N}vP_{v}\left(v\right)}{\sum_{v=1}^{N}vP_{v}\left(v\right)}$$

where *v* is the length of the vertical line, and *v*_*min*_ is the minimum length of the vertical line.

*Trapping Time (TT)* quantifies the mean value of fixed events in each time interval of recurrence. This indicates fine-scale irregularity; therefore, it is associated with determinism and stochasticity.


(9) $$TT=\frac{\sum_{v=v_{min}}^{N}vP_{v}\left(v\right)}{\sum_{v=v_{min}}^{N}P_{v}\left(v\right)}$$

*The maximum vertical length (Vmax)* quantifies the maximum vertical length, whose meaning remains unclear, but it can be related to states with low variability, being locked at a single event:


(10) $$V_{max}=\text{max}\left(v\right)$$

*Time Type Two (T2)* quantifies the average time necessary for a given event to return to its origin point (arbitrarily close to ε) in the phase space. This indicates signal dispersion and, therefore, is associated with stochasticity and chaoticity.


(11) $$\begin{array}{c} T_{k}^{\left(2\right)}\\ =<j_{k+1}-j_{k}>,\text{with}{ℛ}_{i}=\left\{x_{\mathrm{j1}},x_{\mathrm{j2}},\ldots |R_{i,\mathrm{jk}}=1\right\},\forall i,j,k \end{array}$$

### Statistics

We applied the Kolmogorov-Smirnov normality test to all sample data, and because samples could not be considered from a normal distribution, non-parametric tests were used (Massey, 2017). Group differences were assessed using cluster analysis and the Kruskal-Wallis test, followed by the Tukey-Kramer *post hoc* test (Kramer, 1956). For two-sample comparisons, we used the Mann-Whitney test. A multifactorial analysis, clustergram, was applied to choose which RQA metrics would be optimal to distinguish both groups. For this analysis, the Euclidean distance was used to quantify the similarities among the RQA coefficients considering each condition (6-OHDA and sham). The significance level for all statistical analyses was established when *p* < α, where α = 5%, represented in figures by the symbol “*”. All analyses were performed using the MATLAB^®^ software (v. R2016a).

### Principal Components for Clustering Analysis

To highlight the global covariations and similarity effects from the different analyses (RQA on aECG and RQA on ECG animal models) considering the four different conditions LQT, AFR, NSE, and DET, when subjected to different noise levels and different LF/HF ratios, we performed a principal component analysis (PCA). For PCA, one matrix of 84 rows and 10 columns comprising four matrices of 41 rows (representing all 41 signal epochs of 60 s) and 10 columns (representing all RQA factors): one for LQT condition (41 × 10), another for AF condition (41 × 10), another for the NSE condition (41 × 10), and one for the DET condition (41 × 10), each column representing an RQA factor was measured. Therefore, these RQA factors were considered as PCA features and aECG conditions (LQT, AFR, NSE, and DET) as trial effects. This method was repeated for each noise level and LF/HF conditions (Figure 1H).

**FIGURE 2:**
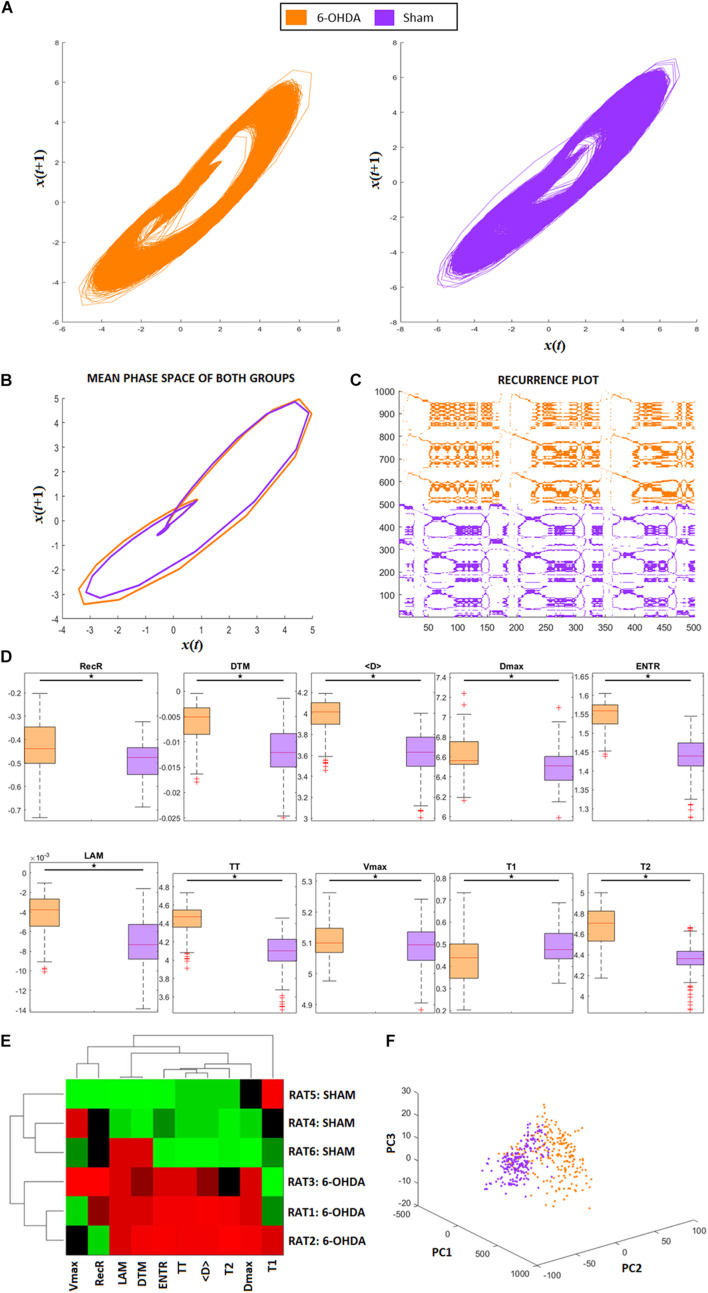
RQA from animal model ECG recordings. **(A)** Phase space trajectories of ECG signals. **(B)** Phase-space mean trajectories related to both groups. **(C)** RP from the ECG signals of both groups. The 6-hydroxydopamine (6-OHDA) signals are more recurrent than ECG from the sham group. **(D)** Statistical comparison between each RQA factor from 6-OHDA and sham groups. All factors are revealed to be statistically different (exhibited in logarithm scale). **(E)** Clustergram of RQA factors using Euclidian distance as the metric of similarity. Each group presents its own set of RQA coefficient, forming two different clusters, 6-OHDA are rats 1–3, and sham are rats 4–6. In this case, Time type 2 (T2), Shannon entropy (ENTR), Trapping Time (TT), mean diagonal length (<D>), and maximum diagonal length (Dmax) are the main coefficients to distinguish one group from the other. **(F)** Principal Component Analysis from RQA factors also reveal that both groups differ from each other, with two distinct clusters with minimal overlap between them.

For all PCA spaces analyzed here, we use only the first two components, PC1 and PC2 (Figures 1I,J), since they were able to explain more than 80% of the data covariances (Figure 2F uses three PCs only for a better visualization).

Finally, all RQA data from the animal models were projected onto the RQA space previously obtained from the aECG conditions through the PCA approach. Once an ECG pattern was projected onto the aECG PCA space, the Euclidean distance was calculated considering each centroid cluster related to each aECG cluster, including the sham group.

## Results

To show the possible differences using traditional coefficients (such as SDRMS) and RQA, we first evaluated the dynamics associated with the four artificial ECG patterns by varying the percentage of noise on their HRV with four different LF/HF ratios. This reveals how the traditional techniques are unstable under different conjugation of noise and LF/HF imbalance. By applying RQA to these aECG signals, we were able to identify and quantify non-linear features associated with each cardio/autonomic condition. In our results, the RQA technique has shown to be more robust and sensitive for detecting autonomic dysfunction under different noises conjugated with LF/HF imbalances on the aECG signals. We were able to evaluate HRV and heart dynamics simultaneously with a better resolution across different temporal scales and under different autonomic-heart conditions.

### Artificial Electrocardiogram

**FIGURE 3:**
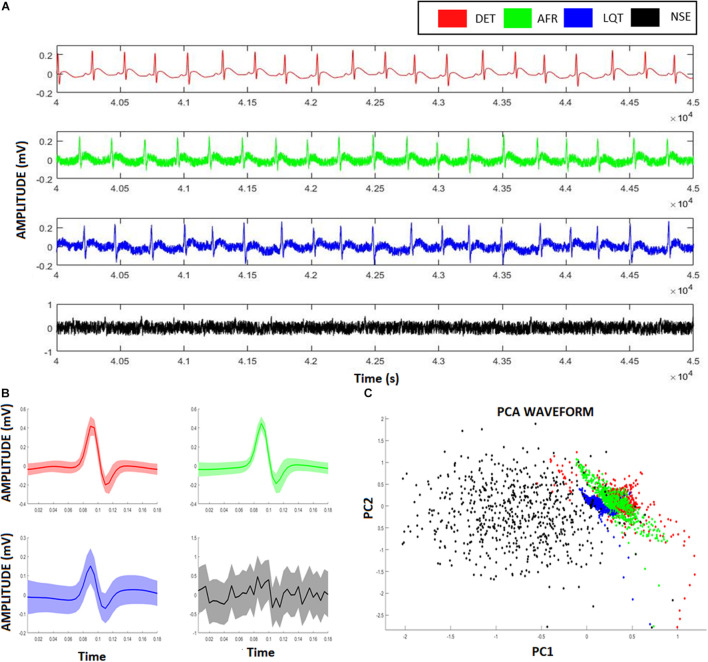
Artificial ECG patterns. **(A)** Red signals represent the deterministic aECG, the green signal represents the atrial fibrillation (AF) pattern relative to the aECGs, the blue signal represents the long-QT (LQT) syndrome pattern relative to the aECGs, the black signal represents a deterministic aECG signal embedded of high noise (NSE). **(B)** The correspondent PQRST-complexes from each aECG model, showing their average and confidence interval. **(C)** Scatter-plot relative to the two first scores from a Principal Component Analysis (PCA) considering all four ECG-complexes shown in **(B).** Despite the clusters formed from different groups overlaps each other, it is still possible to identify and distinguish the groups from their ECG-waveforms.

Figure 3A exhibits the electrographic traces related to the four aECG signals, namely, AF, LQT, DET, and NSE. From their PQRST-complexes, using R-peak as reference to centralize them, they were superposed, and then their confidence interval was calculated (Figure 3B). By using PCA, we were able to separate each group according to their waveform patterns (Figure 3C). This result suggests that their waveforms contain distinct information associated with each heart condition. However, despite this distinguishability, this technique does not provide access to the dynamics of the autonomic system.

**FIGURE 4:**
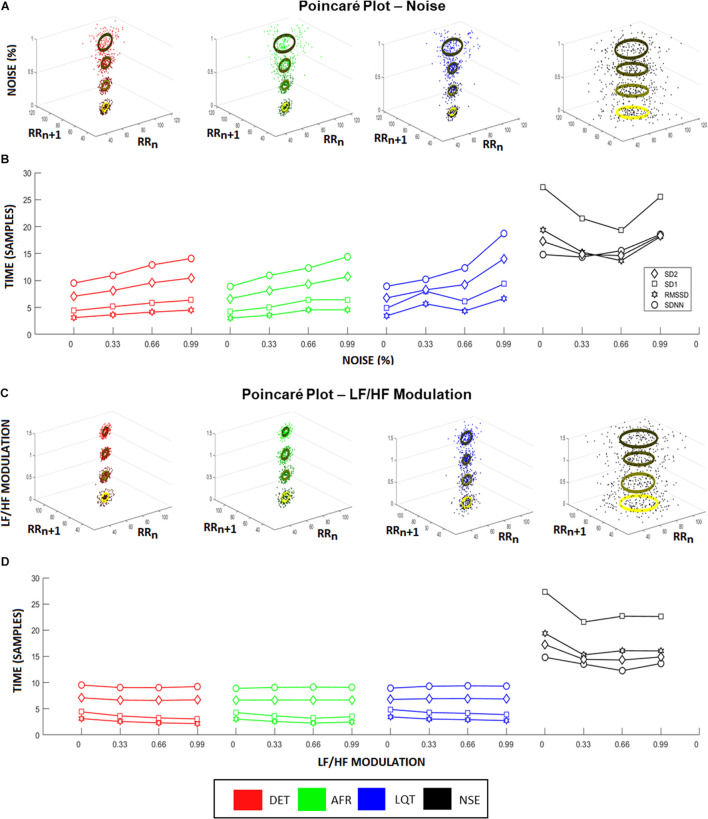
Heart rate variability (HRV) coefficients from aECG signals. **(A)** Higher noise levels spread the Poincaré Maps, raising both, SD1 and SD2, for all groups except noisy ECG signal (NSE). This raise is highlighted in **(B)**, where it represents the changes in HRV characteristics according to different noise levels. In this situation, all coefficients raise linearly according to the increasing of noise levels, where RMSSD showed to be the more sensitive coefficient. **(C)** Changes in Poincaré plot according to LF/HF modulation is observed. In this picture, the colors represent the different intensities of LF/HF modulations. Higher LF/HF values produce higher radius values of Poincaré Map ellipses, emphasizing the differences between SD1 and SD2 coefficients for every group. **(D)** It shows the HRV coefficient values variating according to different LF/HF ratio values. Long-time coefficients, SDNN and SD2, become stable as short-time coefficients, SD1 and RMSSD, decrease their values.

To provide a complementary perspective on ANS dynamics, we also applied Poincaré maps to the aECGs to evaluate their behavior and their relationship to the different LF/HF ratios and noise intensities. Figure 4A shows how Poincaré maps of aECG conditions (*xy*-axis) vary according to their level of noise (ellipses on *z*-axis). This shows that the higher the magnitude of noise applied to the tachograms, the greater the data dispersion. Figure 4B shows the sensitivity of the standard HRV coefficients (SDNN, RMSSD, SD1, and SD2) under different noise levels. Once again, higher values of noise applied to tachograms yielded higher values of HRV coefficients, except for the NSE group, which exhibited a parabolic fluctuation but with indistinct values among the coefficients. Figure 4C shows the opposite effect, focusing on how Poincaré Maps vary, for each aECG condition (*xy*-axis) as a function of LF/HF modulations (*z*-axis). Now, the variation in LF/HF modulations produces subtle restrictions on the ellipsis-shaped limits. Figure 4D shows how HRV coefficients vary as a function of LF/HF modulations.

**FIGURE 5:**
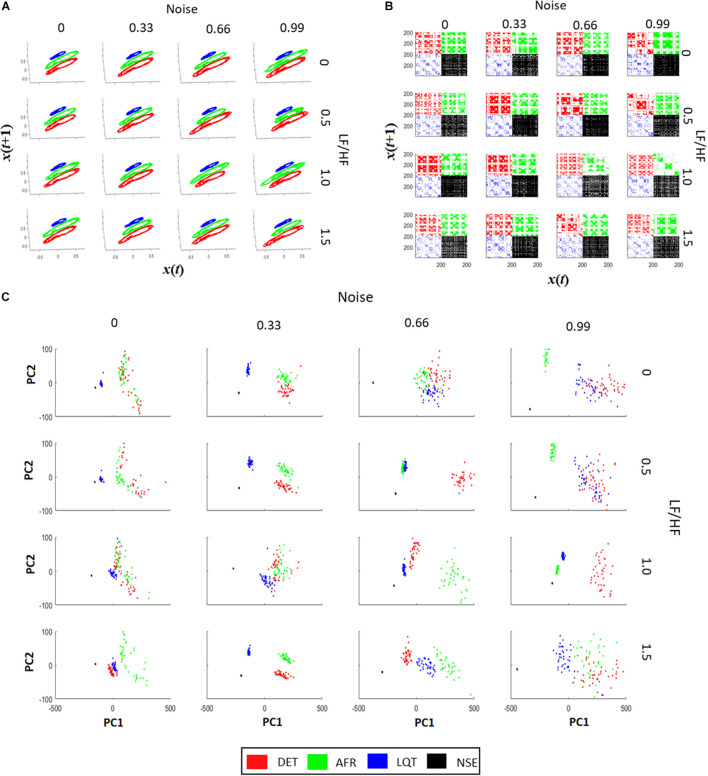
Recurrence quantification analysis (RQA) characteristics of aECG signals. **(A)** The signal recurrences of each group in each condition. Higher values of LF/HF increase the variability of ECG in the phase-space trajectories. **(B)** Each recurrent plot (RP) of each group are different. The changes happen not only on individual dots, but also on the dot-lines and diagonal dot-structures, changing the main characteristics gather from RPs. By increasing the intensity of noise in the tachograms, it turns the RP pattern less periodic, affecting the main structures related to trajectory recurrences in RPs. The LF/HF modulation turns the dot-structures less regular, also affecting the value of dot-structures related to recurrences. **(C)** PCA projections using RQA coefficients (RR, DTM, <D>, Vmax, ENT, TT, T2) for each aECG condition. In most cases it is possible to differentiate each cluster from each group. The raise of LF/HF modulation changes the clusters from Long QT syndrome (LQT) and atrial fibrillation (AF), and the raise of noise, let the RQA features less condensed, making it more difficult to differentiate AF from LQT.

Figure 5A shows all phase-spaces associated with the aECG patterns considering different regimes of noise and HRV LF/HF ratios. LQT (blue) has lower amplitude variation than other groups, decreasing its structure size in comparison to the other groups. For almost all cases, AF and DET presented the same information, distinguishing only in their P-wave structure, which is more visible in DET than AF.

From Figure 5B, it is possible to see that when noise and LF/HF increase, there are more irregular diagonal dot structures on the RPs. In this regime, all four conditions (DET, LQT, AFR, and NSE) present larger and longer periods of recurrence, and still exhibit a trend. The RP vertical line structures are also influenced by noise and modulation, meaning that the ECG signals increase spurious recurrences. When the LF/HF modulation increases, the diagonal structure sizes become irregular owing to the fluctuation of the instantaneous heart rate. These changes represent irregularities in ECG events that reflect variations in HRV. Conversely, when noise increases, the periods of the diagonal structures of the RP are irregular but different from the LF/HF modulations, and the ECG events do not exhibit a trend. This reflects the irregularities of the ECG PQRST complexes, which decrease the recurrences and shorten the rectangles in RP.

Figure 5C shows four distinct clusters for almost all regimes of noise and LF/HF ratios. This means that RQA non-linear features, which represent aECG recurrence over time, are more significant than complex morphological changes in autonomic dysfunction. It is also possible to see that the increase in noise level yields an increase in the cluster size, mixing them.

### Application of Recurrence Quantification Analysis to the Electrocardiogram of Parkinson’s Disease Animal Models

To show the reliability of RQA to real ECG signals, we applied it to a set of ECGs recorded from animal models of PD (6-OHDA) and compared their non-linear features with a sham group and with the four artificial ECG signals. By comparing the RQA coefficients from the real and artificial signals, we were able to distinguish PD ECG features from sham ECG features more effectively than by using ECG waveforms and traditional HRV factors.

**FIGURE 6:**
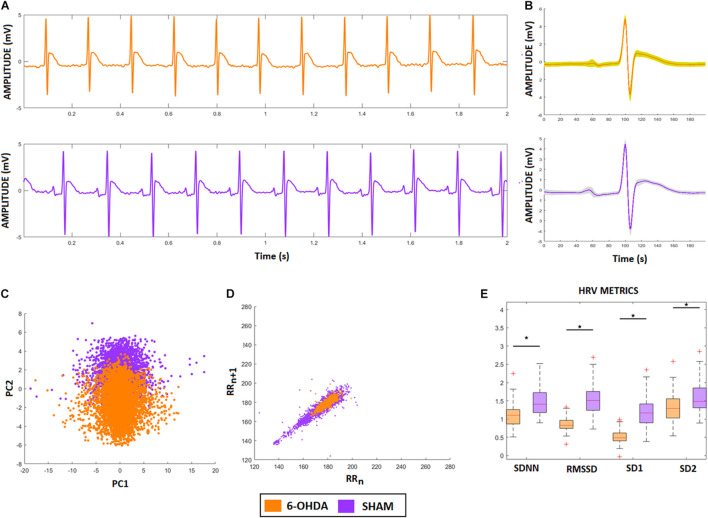
ECG and HRV standard features from animal model. **(A)** ECG signals from both animal models, 6-OHDA in orange and Sham in lilac. **(B)** Morphologic differences of PQRST-complexes. **(C)** Principal Component Analysis of PQRST complex, it is possible to see two clusters, one in orange and one in lilac. **(D)** Poincaré plot of both ECG recordings, showing that 6-OHDA group has a denser cluster. **(E)** HRV coefficients for each group, all characteristics presented significative difference (ρ < 0.05), by comparing 6-OHDA with Sham groups.

Figure 6A shows two representative ECG signals recorded from a 6-OHDA animal model (orange) and from a SHAM animal (lilac). Figures 6B,C show the ECG-complex waveforms for both groups and their PCA, respectively, highlighting the differences between ECG waveforms from the 6-OHDA and sham groups. It is possible to identify two clusters with an overlap, indicating that PCA was not able to detect statistical differences between the ECG complex shapes from 6-OHDA and SHAM groups. Figure 6D displays the clusters from Poincaré mapping associated with the ECG-tachograms from both groups. It shows that the 6-OHDA group has a denser cluster than Sham group, but both present a central dispersion tendency onto the identity line. This effect occurs since their window samples are presented R-R intervals do not exhibit drastic variations over time (such as arrhythmia). Their averages are mainly stationary, yielding this similarity with the main diagonal. These differences were detected through the SD1 and SD2 coefficients, and SDNN and RMSSD, which are different for both groups (Figure 6E). It also shows that for all HRV factors, 6-OHDA groups exhibit significant lower values, indicating that both groups are different in their HRV variability but not necessarily in their ECG complex waveforms.

For these groups, these classic HRV features were already enough to detect the effects of PD on HRV patterns. However, since we cannot properly distinguish their ECG features, the possible effects from isolated cardiac dysfunction could be masked. By applying RQA we can calculate other ECG features that highlight differences that the standard techniques cannot. As we will show, they are more sensible and robust under noise and LF/HF modulations.

Figure 2A shows the phase-space of the signal embedded dimension for both groups (6-OHDA and SHAM), highlighting their recurrences in time. It is possible to see that the SHAM group has more variance along cycles than 6-OHDA group. The mean recurrences relative to each group can be seen in Figure 2B. Even though the ECG recordings have complex morphological changes (as shown in Figures 6A,B), their amplitude changes do not affect the recurrences, meaning that their single event diameters in the phase-space are not different on average, with minor changes along QRS complexes. A representative RP for both groups is exhibited in Figure 2C, where it is shown that 6-OHDA ECG recordings are more recursive than sham ECG recordings. These recurrences are evaluated through the RP diagonal lines that reflect the recurrences of their PQRST complexes, and through the RP vertical lines that reflect large-scale temporal recurrences.

In Figure 2D, we can compare each RQA factor, all of which reveal significant differences between the 6-OHDA and sham groups. Except for factor T1, group 6-OHDA exhibited higher values. Considering all RQA factors, we conclude that the 6-OHDA group presents signals that are less stochastic and less chaotic than those of the sham group.

The results are presented in Figure 2E corroborate the statistical pairwise comparisons shown in Figure 2D, considering a clustergram analysis for both animal groups and all RQA factors as markers. It can be seen that seven RQA factors can distinguish both groups, in this case: LAM, DET, T2, ENTR, TT, < D >, and Dmax. These factors indicate that the main changes in 6-OHDA ECG signals in comparison with SHAM are more relevant in their level of periodicity (LAM, TT, T2) and in their waveforms (DET, ENTR, <D>, and Dmax), as shown in Figure 2D. That is, the 6-OHDA group PQRST complex events stay for longer periods of time in a single event than the SHAM group, and it takes more time to return to a single event. This feature can express bradycardia and lower variability in RR intervals. Furthermore, the 6-OHDA amplitude values tended to remain stable over time, with lower variability in signal amplitude.

In Figure 2F, we see the PCA analysis using RQA factors as PCA features. It is possible to completely distinguish 6-OHDA from the sham group using RQA factors, corroborating previous results. In contrast to Figure 6C, which considers only waveforms, RQA factors simultaneously capture ECG features and HRV.

These analyses show how RQA can be used to detect other statistical signal features related to comorbidities that standard techniques cannot detect, mainly under noise and LF/HF modulations.

### Recurrence Quantification Analysis Cluster Analysis: Artificial Electrocardiogram vs. Electrocardiogram

To quantify possible similarities between real ECG features with aECG features, we projected the RQA factors related to 6-OHDA and sham groups onto the PCA space constructed by the four RQA factors calculated from aECG (DET, AFR, LQT, and NSE). Additionally, the Euclidean distance was calculated between each of the five centroid clusters (DET, AFR, LQT, NSE, and sham), using the 6-OHDA centroid cluster as the main reference. These metrics were chosen since we wanted only to quantify the proximal clusters, and the physiological features attributed to the aECG were simplified in comparison to the real ECG. By adopting more rigorous metrics of similarity, we were not able to evaluate the cluster correspondences.

**FIGURE 7:**
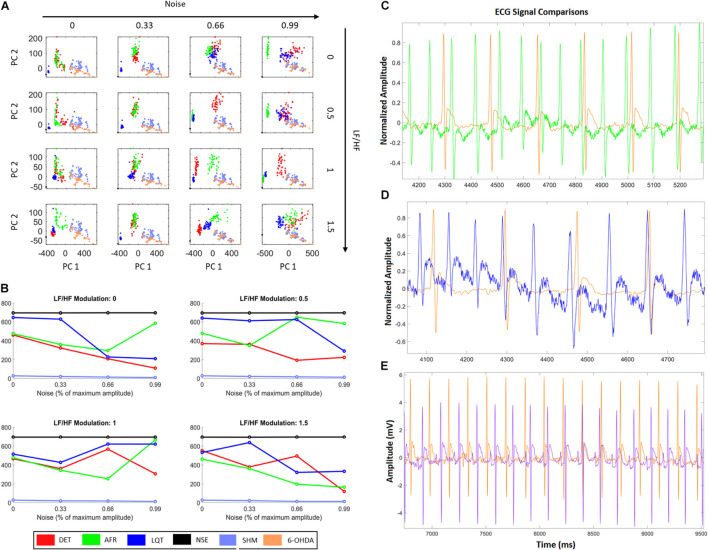
HRV characteristics combined with RQA metrics from aECG and ECG animal models. **(A)** Projections of RQA factors calculated from the animal ECGs onto the principal components space constructed from the RQA factors calculated from the four aECG patterns. **(B)** The distances calculated from all centroid clusters in relation to the 6-OHDA centroid cluster, considering the different conditions in terms of HRV modulation and noise intensity. The closest cluster (related to real ECGs) are the cluster from DET aECG pattern for all conditions of HRV and noise. There is an alternation of the distance values, that occurs mainly for high levels of noise, where atrial fibrillation (AF) and Long-QT Syndrome (LQT) change their centroid values. It indicates that high noises in tachograms could interfere in standard metrics. **(C)** Comparison of AF to 6-OHDA for LF/HF modulation equal to 1.5 and noise equal to 99%. Both signals look similar, where their centroid cluster distances exhibit the lowest distance values. **(D)** It shows an example of LQT aECG pattern considering a noise intensity equal to 99% and LF/HF modulation equal to 0, compared with 6-OHDA ECG signal. The proximity of P-waves and T-waves suggests all signals exhibit only QRS-complexes and one bi-event defined by the union of P and T waves. **(E)** Differences between 6-OHDA and Sham ECGs.

From Figure 7A, it is possible to see that additional external noise on signals yields dispersion, making it difficult to distinguish which group is nearest to 6-OHDA. Figure 7B shows how the distance values from the 6-OHDA cluster vary according to each noise and LF/HF modulation. It can be seen that the 6-OHDA RQA factors stay nearest to the AFR for higher LF/HF values and nearest to DET for lower LF/HF values. This indicates that the 6-OHDA ECG patterns exhibit AFR features. Figure 7C shows the ECG recordings corresponding to the last square values shown in Figure 7B, with 99% noise and 1.5 of LF/HF modulation. Figure 7D shows the ECG recordings corresponding to the second square of Figure 7B, with 99% noise and 0.5 of LF/HF modulation.

## Discussion

In this work, we propose a proof-of-concept study showing that using only linear properties of ECG recordings may be insufficient to describe integrally cardiac-autonomic dysfunctions. Instead, we use the RQA technique that intrinsically incorporates temporal event recurrences at different time scales, which can consider heart and autonomic conditions simultaneously. Through a simplified computational model, we were able to build two cardiac/autonomic conditions (and two other controls) that commonly can be detected on the morphology of ECG-complexes and on their HRV, mainly in PD. In sequence, we calculated the standard temporal HRV factors SDNN and RMSSD, SD1, and SD2 coefficients, and applied PCA to their corresponding waveforms to search for different physiological ECG patterns. However, neither PCA nor HRV factors were able to properly distinguish the variations associated with each condition (especially, atrial fibrillation and long-QT syndrome). By varying the noise intensity and autonomic LF/HF ratio associated with their tachograms, we could compare the sensitivity and robustness of the standard HRV factors.

We verified that all HRV characteristics increased linearly as noise intensity increased (Figure 4C), indicating that HRV factors can be disrupted by environmental noise, outliers, or misdetections. The same effect was observed within Poincaré plots, whereas the intensity of noise increased the scattering as well (Figure 4A). However, even with these effects, none of the coefficients was sensitive enough to properly detect noise and LF/HF modulations. The SDNN and SD2 factors, for instance, remained stable for every LF/HF value, and RMSSD and SD1 decreased their values only slightly. These results could be due to the short term of aECG signals since they were simulated only for 5 min (Shaffer et al., 2014). However, this means that, at least, these coefficients always require long ECG recordings to operate properly.

RQA was more sensitive and robust in detecting non-linear features related to each of the four conditions expressed on their aECGs. We calculated nine RQA factors associated with the four aECG conditions: DET, AF, LQT, and NSE (Figure 5A). We verified that as the noise intensity increased, the RPs also increased their number of dot structures, and the number of spurious correlations increased (correlations of dot length lower than three; Figure 5B). We can see that the periodicity of the ECG signals is strongly modulated by the LF/HF ratio changes (Figure 5C). As the LF/HF ratio increases, the RPs lose vertical dot structures, indicating an increase in stochasticity (Garfinkel et al., 1997; Schauerte et al., 2001; Chen et al., 2014). It is important to mention that a healthy heart does not exhibit a purely deterministic dynamic. In fact, it presents stochasticity given by internal and external factors, and non-periodicity is reported, as chaotic dynamics, which makes the RQA a better technique to evaluate it (Braak et al., 2007; Pyatigorskaya et al., 2016; Çanga et al., 2018).

When we analyzed the RQA factors (RecR, DET, LAM, TREND, T2, ENTR, TT, < D >, and D_max_) as PCA variables, we were able to explain all the covariations associated with each specific group under all different noise and HF/LF modulations (Figure 5C). This indicates that RQA metrics are not only more sensitive for detecting noise and autonomic modulations on short-time ECG recordings, but can also explain HRV conditions more accurately.

### Application: Electrocardiogram From Animal Models of Parkinson’s Disease

By applying PCA to the ECG complex waveforms recorded from animal models, we verified that they were not able to completely distinguish 6-OHDA from the SHAM groups (Figure 7E). This lack of difference in ECG signals gave rise to two questions: (i) Are RQA and/or Poincaré Map factors capable of differentiating each one of these conditions?, and (ii) How do HRV features influence each condition?

To answer these questions, we first analyzed different features related to heart rate variability (HRV) time series considering each ECG pattern, associated with 6-OHDA and sham groups, looking for distinct sympathetic (SNS) and parasympathetic (PNS) activities. Using Poincaré Maps and standard factors (SDNN and RMSSD), we verified that the 6-OHDA group had lower HRV coefficients than the sham group (Figures 6D,E). This result can also be verified through the smaller cluster of 6-OHDA group in the Poincaré map (Figure 6D), which comes from the low variance of R-R time sizes, which is associated with its higher average value (cluster centroid) in comparison to the SHAM group. Therefore, the 6-OHDA group had longer RR intervals, indicating bradycardia and decreased sensitivity to fast responses to external stimuli (Pursiainen et al., 2002; Imrich et al., 2008). According to the literature, one possible explanation for this low HRV activity in PD is a lack of sympathetic activity response (Goldstein et al., 2000).

In terms of RQA, the ECG recordings from the 6-OHDA group were more recursive than the ECG signal from the sham group, since its RecR and DTM factors were higher than those of the sham group. This result indicates that the 6-OHDA group had a lower ECG amplitude variability over time, with more recurrent events. This can be checked through its phase space (Figure 2A) and Poincaré map (Figure 6D). These effects could be physiologically interpreted mainly by: (*i*) low concentrations of Ca^+2^ in conductive heart cells (Kramer, 1956); (*ii*) myocardial changes that lead to atrial fibrillation and yield a periodicity increase of specific events along the ECG signals (Hong et al., 2019); and (iii) heart sympathetic denervation, which decreases HRV and produces regular and periodic ECG (Orimo et al., 1999; Goldstein et al., 2000).

The higher values of < D > in 6-OHDA ECGs compared with sham indicates that their signals present higher self-similarity, which is also revealed by their event periodicities. This RQA factor indicates that the ECG signals have small perturbations across time, with one major divergence found in the QRS complex. Additionally, higher values of Dmax, TT, LAM, and T2 in 6-OHDA signals compared to sham signals indicate that their ECG events are more recurrent over time. That is, given an ECG-reference, such as R-pick, the way it appears along the signal in time is more regular and similar to the previous ones. While Dmax and LAM coefficients exhibit a more deterministic dynamic, hinting at a low autonomic modulation (in both the PNS and SNS branches), TT and T2 coefficients indicate that ECG events are more alike and longer. Long events come from low amplitude changes and represent more “rigid R-R intervals,” corroborating Poincaré maps (Marwan et al., 2002; Marwan, 2003, 2006). Furthermore, a decrease in heart rate tends to increase the periodicity of the signal, which can be promoted by PD bradycardia (Buob et al., 2010).

Higher values of ENTR indicate that the system itself has some variation in amplitude and is more complex. In this way, the values of ENTR for 6-OHDA ECG recordings are the first indication of greater chaoticity than those of sham signals, in the sense that they have some physical constraints that modulate its activity. This chaoticity is also justified by considering the values of Dmax and TT of 6-OHDA (Wolf et al., 1991; Shibata et al., 2009). One possible origin of this physical constraint is the potential fluctuations due to atrial fibrillation (AF), caused by the decomposition of P-waves into many short waves (Goldstein, 2006).

The coefficients Vmax and T1, despite presenting a significant difference, did not help to discriminate the PD condition, using 6-OHDA models, from sham as revealed by the Clustergram analysis (Figure 2E). Using the seven RQA factors indicated by the Clustergram (< D >, D_*max*_ ENTR, TT, LAM, DTM, and T2) as PCA features, this result can be corroborated. Furthermore, Figure 2F shows that by using the factors, the differences between both groups are optimized and clarified, indicating once again that RQA coefficients explain the variations between both groups.

Considering all RQA coefficients together, we can conclude that ECG signals from 6-OHDA animal models exhibit higher irregularity in their morphology over time, including higher variation in its amplitude, but lower variation in its R-R intervals. This result is probably a consequence of the effect of Parkinson’s disease on autonomic control, which promotes a self-paced rhythm with a strong interference of the parasympathetic path that decreases HRV (Shaffer and Ginsberg, 2017). This excessive regularity makes the heart less flexible to changes, which may be one of the causes of orthostatic hypotension (Smit et al., 1999; Velseboer et al., 2011). Although RQA exhibited pairwise statistical differences, when applied to 6-OHDA and SHAM groups, it was still missing a proper physiological interpretation associated with a more global and integrated analysis of these factors, mainly for specific heart and autonomic conditions related to PD, such as atrial fibrillation and long Q-T.

Finally, we projected all RQA factors calculated from the ECG animal models, 6-OHDA and SHAM, onto the RQA PCA space calculated from the four artificial ECG conditions (DET, AF, LQT, and NS). With these projections, we quantify which of the four artificial conditions the 6-OHDA group would statistically resemble. In this way, by evaluating the centroid distances from each aECG condition to the 6-OHDA conditions, we saw that the sham cluster was the nearest group and the NSE cluster was the furthest. Furthermore, for high noise intensity (0.99), independent of the autonomic frequency ratio, the DET and AF clusters were the closest clusters. This result is expected because aECG models are limited and real ECG signals have complex components (beyond noise and modulation) that differentiate both from all aECG patterns.

Nevertheless, by looking at AF and LQT clusters (green and blue), we see how their distances to 6-OHDA vary according to different noise levels and autonomic modulations. For all LF/HF ratios, the LQT clusters became closer to 6-OHDA for a higher level of noise. This effect can occur because of the addition of noise to the aECG tachograms, which may superpose T- and P-waves, as shown in Figures 5A, 6C–E. Conversely, AF clusters are closer to the 6-OHDA clusters for high values of LF/HF modulation. This can be seen by comparing their ECG morphologies, as shown in Figures 6C–E. Both signals, aECG AF and ECG 6-OHDA, present no P-waves. This increases the R-R intervals, which are expressed by the large vertical lines in their RP (Figures 4B, 6C).

Although the metrics and model features under this quantification were simplified (for human beings all parameters used to construct the aECG must be readjusted), these results suggest that 6-OHDA signals could be correlated with AF signals, indicating a possible tendency of this pathology in PD (van Dijk et al., 1993; Velseboer et al., 2011). Additionally, these results also suggest that LF/HF modulation could lead to misinterpretations due to changes in the morphology of ECG signals, making it difficult to distinguish all groups from low noise values. The main differences between groups were found for intermediate values of LF/HF modulation, where the main changes in all groups occurred, making it easier to pinpoint the cluster condition closest to the 6-OHDA group. A previous study has already indicated that AF is possibly related to autonomic imbalance, especially when one of the branches is highly activated in relation to others (Marwan, 2003).

It is important to note that the addition of noise in the aECG tachograms may modify their aECG morphologies, which may be a limitation of the model. Furthermore, any alteration of heart calcium flux may also lead to an alteration in the myocardial dynamics itself (Ren et al., 2004). These changes also affect heart waveform dynamics, thereby promoting a possible misinterpretation of HRV factors. Therefore, despite showing that RQA factors are more sensitive to ECG and tachogram dynamics, it is still necessary to know if there are well-defined signatures associated with ECG changes caused by a lack of autonomic modulation or by a lack of myocardial response. Both causes can be interpreted as dysautonomia, and the source of the autonomic imbalance (Shibata et al., 2009; Buob et al., 2010) is not obvious from the ECG. However, we emphasize that this issue is a general limitation for HRV analysis that uses only ECG signals, and through RQA factors, it is possible to assess not only “static pictures” of the ECG signals or global coefficients (as averages and deviations along time) but also a reflection of the recurrences and periodicities of the signals (Shaffer and Ginsberg, 2017).

In summary, the artificial ECG patterns associated with RQA factors can be a new approach to help understanding the complex dynamics found in real ECGs. Here, despite the simplifications, the RQA factors suggested that the 6-OHDA group can present atrial fibrillation (AF) mainly for high values of LF/HF ratios. It also pointed out that 6-OHDA ECG recordings exhibit high variations in baseline and temporal regular events. These features have been associated with reduced cerebral blood flow and, recently, a high risk of sudden death from epilepsy (Billeci et al., 2018; Khazaei et al., 2018).

This proof-of-concept study indicates that by applying RQA technique on ECG signals from PD of animal models, they present lower variability in periodicity (RR intervals) but higher complexity in their baseline (amplitude). This means that the 6-OHDA ECG signals exhibit more deterministic event intervals but more stochastic traces. Most likely, these characteristics suggest a lack of autonomic modulation. We interpret it as an “uncalibrated ANS” where both branches work together, but the relationship between them is no longer cooperative, producing an unbalanced heart dynamic. Although the application study was limited due to the small animal samples, we believe this work opens a new direction for the application of RQA to ECG signals. RQA is a very promising technique to advance new studies of heart-brain dynamics to elucidate other heart or autonomic changes in PD patients.

## Data Availability Statement

The raw data supporting the conclusions of this article will be made available by the authors, without undue reservation. Requests should be made to to jean.faber@unifesp.br.

## Ethics Statement

All the experiments were approved by the Animal Care and Use Committee of Ethics of the Federal University of São Paulo (protocol: CAAE 6463110417), and the analysis applied on biological signals were approved by the Ethics and Research Committee of the Federal University of São Paulo, under the protocol number: CAAE 7299310719.

## Author Contributions

LS, LD, LF, FS, and JF contributed to investigation. LS, SC, and JF contributed to methodology. SC, CS, and FS contributed to conceptualization. LS contributed to data curation, formal analysis, and visualization. FS and JF contributed to validation, project administration, supervision, and contributed to funding acquisition. LD and LF contributed to resources. LS and JF contributed to software. LS and RC contributed to writing—original draft preparation. LS, LD, LF, RC, CS, FS, and JF contributed to writing—review. All authors contributed to the article and approved the submitted version.

## Conflict of Interest

The authors declare that the research was conducted in the absence of any commercial or financial relationships that could be construed as a potential conflict of interest.

## Publisher’s Note

All claims expressed in this article are solely those of the authors and do not necessarily represent those of their affiliated organizations, or those of the publisher, the editors and the reviewers. Any product that may be evaluated in this article, or claim that may be made by its manufacturer, is not guaranteed or endorsed by the publisher.
